# The Pen Is Milder Than the Blade: Identification Marking Mice Using Ink on the Tail Appears More Humane Than Ear-Punching Even with Local Anaesthetic

**DOI:** 10.3390/ani11061664

**Published:** 2021-06-03

**Authors:** Charlotte C. Burn, Nur H. B. Mazlan, Natalie Chancellor, Dominic J. Wells

**Affiliations:** 1 Animal Welfare Science and Ethics, Royal Veterinary College, Hertfordshire AL9 7TA, UK; nchancellor@rvc.ac.uk; 2 Comparative Biomedical Sciences, Royal Veterinary College, London NW1 0TU, UK; nmazlan@rvc.ac.uk

**Keywords:** 3Rs, animal welfare, anxiety, culture of care, identification marking, laboratory animals, mice, pain, refinement, reproducibility

## Abstract

**Simple Summary:**

Laboratory mice often look identical, so they are commonly marked by cutting the ear via ear-punching, or marking the tail with permanent marker. Ear-punching is permanent but could be painful, and mice could become stressed by weekly tail-marking, so we compared impacts on mice over 5 weeks. We also explored whether local anaesthetic cream could reduce any ear-punching effects. We found that ear-punching, even with anaesthetic, caused mice to be sniffed and groomed by their cagemates for at least 5 min after marking. Mice ear-punched with anaesthetic also groomed themselves and their ears ~5 times more than tail-marked and control mice. Facial grimacing was most common in the unmarked cagemates of tail-marked mice, and possibly in mice ear-punched with anaesthetic. The next day, mice ear-punched with anaesthetic were significantly less likely to eat unfamiliar food in an anxiety test than tail-marked or control mice. Over 5 weeks, ear-punched mice approached the handler significantly less than unmarked mice did, and tail-marked mice showed reduced defecation during re-marking. Other behaviour, bodyweight, and corticosterone showed no treatment effects. This suggests ear-punching caused some signs of pain and anxiety, and anaesthetic did not help. Tail-marking appeared more humane, showing no differences from the controls.

**Abstract:**

Identification marking mice commonly involves ear-punching with or without anaesthetic, or tail-marking with ink. To identify which is most humane, we marked weanling male BALB/c mice using ear-punching (EP), ear-punching with anaesthetic EMLA^TM^ cream (EP+A), or permanent marker pen (MP). We compared marked mice, unmarked cagemates, and control mice (*n* = 12–13/group) for 5 weeks, reapplying MP weekly. Treatment-blind observations following marking showed that EP and EP+A mice were allogroomed (*p* < 0.001) and sniffed (*p* < 0.001) by their cagemates more than MP and control mice were. EP+A mice groomed themselves (*p* < 0.001) and their ears (*p* < 0.001) ~5 times more than most other mice; their cagemates also increased self-grooming (*p* < 0.001). Unmarked MP cagemates (*p* = 0.001), and possibly EP+A mice (*p* = 0.034; a nonsignificant trend), grimaced the most. The following day, half the EP+A mice showed hyponeophagia versus no MP and control mice (*p* = 0.001). Over the 5 weeks, EP mice approached the handler significantly less than unmarked cagemates did (*p* < 0.001). Across weeks, defecation during marking of MP mice decreased (*p* < 0.001). Treatment showed no effects on immediate responses during marking, aggression, bodyweight, plus-maze behaviour or corticosterone. MP mice showed no differences from controls, whilst EP and EP+A mice showed altered behaviour, so ink-marking may be the more humane identification method.

## 1. Introduction

Many methods for identification marking animals exist, but some have the potential to cause pain or stress [1,2,3,4]. Therefore, refinement of marking methods could improve welfare for large numbers of animals. In the animal research sector, it could also improve scientific reproducibility. A survey revealed that ear-punching and/or -notching (85%, combined) were the most commonly used laboratory mouse identification methods in the UK and Ireland, followed by marking the tail with marker pen (63%) [1]. Similar results were found in an earlier UK survey [5]. In Europe, ear-punching/notching is also the most common method of genotyping [2]. Here we aim to compare the animal welfare effects of ear-punching versus tail-marking with a pen, with a view to refine these common mouse identification marking methods.

### 1.1. Effects of Ear-Punching on Mouse Welfare

The terms ‘ear-punching’ and ‘ear-notching’ are sometimes used interchangeably, and sometimes collectively as ‘ear biopsy’, especially when used for genotyping. Here we refer to ear-punching as the use of a mouse ear-puncher to create circular or semicircular holes in the ear pinna, whereas ear-notching involves cutting v-shapes into the pinna using scissors. 

Ear biopsy by either means could cause stress and anxiety partly due to the restraint required [6,7], especially if lifting mice by the tail [8]. It may additionally cause acute and possibly longer term pain, because it severs sensitive tissues. Most mouse users (70%) surveyed in the UK and Ireland believed it caused only mild pain or stress, whilst 27% suggested a moderate level [1]. Weanling mice ultrasonically vocalised more during ear-punching (31% of mice) than during a sham procedure (8%) [9]. Restraint and ear-punching both raised heart rate and body temperature in male HanIbm: NMRI mice for at least 1 h, but ear-punching seemed to take longer to return to baseline (approximately 3 h for heart rate and 5 h for temperature) [6]. Similarly, in female Swiss–Webster mice, ear-punching (but not restraint) increased both heart rate and body temperature for at least 1 h [10]. Both ear-punching and ear-notching caused mice to suddenly pull their heads away from the handler more than restraint alone, followed by more ear-grooming and freezing behaviour once back in the homecage [11]; also, after 24 h, fewer ear-notched than unrestrained control mice consumed novel food during a hyponeophagia test, suggesting increased anxiety. 

Over the longer term, aggression between ear-notched male C57BL/6 mice was higher than in tail-tattooed ones, as measured via post-mortem lesion scores [12]. Taken together, this literature therefore suggests that there is scope for refining ear-biopsy procedures to reduce pain and/or stress.

### 1.2. Use of Local Anaesthetic for Identification Marking

Ear biopsy is usually performed without analgesia or anaesthesia [6,13,14], especially if such substances could interfere with experimental purposes. However, where experimental aims allow, this is a promising route for refinement. For example, rabbits undergoing ear tattooing with EMLA^TM^ cream (AstraZeneca, Luton, UK; a local anaesthetic containing lidocaine and prilocaine) [15], applied 20 min previously, showed significantly smaller increases in heart rate and blood pressure, less struggling and vocalisation, and lower Rabbit Grimace Scale scores during the procedure than controls [16]. EMLA^TM^ was similarly effective in reducing pain responses to venepuncture in rabbits, cats and dogs [17]. In contrast, EMLA^TM^ cream is yet to show any efficacy for reducing pain during venepuncture or tail-biopsy in mice [18,19,20] and rats [17]. However, the efficacy of EMLA^TM^ cream for reducing pain from ear-punching in mice has not previously been investigated. 

### 1.3. Use of a Marker Pen for Identification Marking

Marking the fur or tail using a pen could avoid potential pain and stress from ear-punching (as long as genotyping is not required, because ink marking does not involve any tissue removal). Tail-marking with a pen was rated by 82% of UK and Ireland survey respondents as being only mildly stressful to mice [1]. However, rats avoided the odour released from uncapped marker pens [21], and BALB/c mice showed similar response when tested in two-choice Grice box [11]. After 1 h of forced inhalation of vapours from uncapped marker pens of seven different (unspecified) types, mice showed respiratory irregularities and behavioural abnormalities, such as tremors and ataxia [22]. However, in practice, mice are only exposed to uncapped pens for a few seconds during marking, and the relatively small volume of ink deposited onto the mouse may dry rapidly, so evaluation of mouse behavioural and physiological responses to a realistic ink-marking procedure is needed.

Another consideration is that ink marks fade with time, even with so-called ‘permanent’ marker, so reapplication is necessary. Repeated marking could increase animal stress, although mice can habituate to repeated handling and restraint (without marking) e.g., [23,24]. Rats tail-marked every 2–3 weeks showed increased harderian gland secretion during handling compared with unmarked cagemates, and yet spent longer on the open arms of an elevated plus maze (EPM), suggesting that tail-marking made them more averse to handling but less anxious generally [21]. Mouse pups marked daily using marker pen also appeared less anxious as adults in an EPM than those unmarked as pups did, but this could be due to the anxiolytic effects of early handling rather than effects of marking per see [25]. The acute and longer term effects of tail-marking on weaned mice have seemingly not been studied previously.

### 1.4. Social Effects of Marking

It is not uncommon to leave one mouse unmarked when identification-marking a cage of mice. However, the consequence of housing marked and unmarked mice together is little known. Alarm pheromones released during stressful procedures can affect nearby mice [26], so perhaps marking could affect pheromone release from marked mice and thus affect unmarked cagemates. Additionally, marking could affect dominance. For example, male BALB/c mice whose fur was marked with hair dye were more frequently subordinate than their unmarked cagemates, showing less aggression and reduced urine counter-marking [27].

### 1.5. Aims and Objectives

We aimed to assess whether use of EMLA^TM^ cream before ear-punching, or use of a marker pen on the tail, could help refine identification marking compared with ear-punching alone. We therefore compared the effects of (a) ear-punching, (b) ear-punching with prior application of EMLA^TM^ cream, and (c) tail-marking using permanent marker pen on signs of pain, anxiety, stress and aggression in marked mice, their cagemates, and controls. 

Specific signs of pain or discomfort that we measured included pulling the head away (flinching) and vocalising during marking [19,28], as well as mark-directed grooming [29] and facial grimacing in the homecage [20,30]. Signs of acute anxiety or fear included defecation and urination during marking [19,28]; hiding, freezing, reduced food/water consumption, and increased grooming in the homecage [31]; and increased faecal corticosterone concentration [32,33]. We also measured longer lasting anxiety via an EPM [19,21,25]; a hyponeophagia test [34,35]; reduced voluntary interaction with the handler [8]. Finally, we observed social effects including aggression and wounding [12,36], allogrooming, and sniffing of the cagemate [31].

## 2. Materials and Methods

### 2.1. Animals and Housing

Male BALB/c mice (from stocks originally purchased from Charles River) were bred in-house. The home room was maintained at 21 ± 1 °C with 41% relative humidity, and was on a 12 h light schedule (7 am–7 pm). Food (RM1 (E), Special Diets Services) and tap water were available ad libitum. Cages and water bottles were cleaned weekly.

At 19–21 days of age, two male littermates at a time were allocated to one of four cage groups: (1) Ear-punch cage (*n* = 13), (2) Ear-punch with EMLA^TM^ cage (*n* = 13), (3) Marker pen cage (*n* = 12), and (4) Control cage (*n* = 12), with every fourth cage being of the same treatment. Each conventional plastic cage (12 cm × 27 cm × 16 cm) contained woodchip bedding, paperwool, and a cardboard tube. Mice were left to habituate to their housing condition for 5–7 days. All animal procedures were performed in accordance with the Animals (Scientific Procedures) Act 1986 under project PPL 70/7514 approved by the Royal Veterinary College Ethics and Welfare committee.

### 2.2. Weighing and Physical Health Checks

Mice were weighed using digital weighing scales 1 day before their treatment, and then 1, 2, 3 and 4 weeks afterwards. To investigate if marking methods would cause negative social interactions, we observed for signs of barbering (whisker loss or bald skin patches), scratches or wounds on each mouse at each of the five time points. 

### 2.3. Treatment Procedure 

One day after the first weighing, the treatments took place during 07:30–10:00 h, with the experiment staggered across 19 days between 23rd July and 15th Oct 2014; 1–5 cages were allocated per day as mice reached 4–5 weeks old. In each cage, one mouse was marked and the other unmarked, with the exception of Control cages in which both mice were unmarked. There were therefore seven different mouse treatments:

- Control;
- Ear-punch (EP);
- Cagemate of EP (C-EP);
- Ear-punch with anaesthetic EMLA^TM^ cream (EP+A);
- Cagemate of EP+A (C-EP+A);
- Marker pen on the tail (MP);
- Cagemate of MP (C-MP).

EP+A mice had ‘EMLA cream 5%’ (AstraZeneca, Macclesfield, UK) applied onto both surfaces of the right ear pinna while in their homecage, 20 min before ear-punching. The cream contained active ingredients lidocaine (25 mg/g) and prilocaine (25 mg/g), with macrogolglycerol hydroxystearate, Carbomer 974P, sodium hydroxide and purified water [15]. An experienced female experimenter (NHM) carried out identification marking in a procedure room adjacent to the homecage room. Mice undergoing identification marking were lifted by the tail-base and placed onto the wire cage lid. EP and EP+A mice were scruffed and held vertically by the handler. A punch was placed centrally into the right pinna using a 2 mm (punch diameter) mouse ear-puncher (Harvard Apparatus Ltd., Cambridge, UK). Tail-marking was performed on MP mice by drawing a 2 mm × 4 mm rectangular mark on the dorsal tail-base using a black Permanent marker pen (Sharpie^TM^ Fine Point, Newell Rubbermaid UK Services Ltd., Lichfield, UK). During marking, the occurrence of pulling the head away (a sudden head movement, pulling away from the handler; EP and EP+A only), audible vocalisation, urination and defecation were recorded. 

Unmarked cagemates were present in the room during marking, but were left unhandled in their homecage, mimicking common practice. Control mice were brought into the procedure room but were left in their cages on the worktop for about 15 s (approximate time to complete marking). For MP mice, tail-marking was repeated weekly four more times and their immediate responses scored each time. 

### 2.4. Homecage Behaviour Recording after the Treatment Procedure

Almost immediately following treatment, homecage behaviour was video recorded for 5 min on the workbench. To view inside cages, most paperwool was removed during recording, but bedding and the cardboard tunnels remained. Videos were later coded using continuous focal sampling using Interact 9 software (Mangold International, Arnstorf, Germany). The ethogram is displayed in Table 1. The observer (NC) who carried out the coding was highly experienced in systematic behavioural analysis. This observer was initially blind to the hypothesis, and remained entirely blind to the EMLA^TM^ treatment, but gradually became aware of the ear- and tail-marks whilst observing the videos. Microsoft Excel’s ‘Randomize’ function was used to determine the order in which videos were watched, and the order in which the two mice were observed per cage was standardised by always starting with the mouse closest to the left-hand side (or the closest, if neither was leftmost). Once the first mouse per cage had been observed, the video was restarted to observe the second mouse.

### 2.5. Elevated Plus Maze

To assess anxiety, EPM testing was carried out for all mice 6 h after treatment, and 4 weeks later. The maze comprised black Perspex and was raised 60 cm above the floor. It had two open arms (30 cm × 5 cm with 0.5 cm high borders), two closed arms (30 cm × 5 cm with 20 cm high walls) and a central square (5 cm × 5 cm). EPM testing took place between 3 pm and 4.30 pm in a procedure room. Before the first test session, to maintain consistent scent profile even at the first testing, an off study male BALB/c mouse was allowed to explore the EPM for 5 min. Between mice, the maze was cleaned with diluted (1:100) Distel solution, followed by water, and then wiped dry using tissue paper. Each mouse was lifted out of its cage by the tail and placed in the central square of the EPM facing an open arm and its behaviour was recorded for 5 min. The number of stretched attend postures, head dipping, urine spots and faecal boli were recorded live by the female observer (NHM), who had marked the mice and who stood quietly 1 m away from the maze. Time spent in the central square, closed arms and open arms was recorded from video by an observer who was blind to treatment, using Interact 9 software (Mangold International, Arnstorf, Germany). 

### 2.6. Hyponeophagia Test 

One day after marking (between 11 a.m. and 12:30 p.m.), a hyponeophagia test was conducted in the procedure room. Two pieces of novel food (Original sweetcorn, Green Giant™, General Mills, Uxbridge, UK) were dropped inside the homecage and the latency for each mouse to begin eating was recorded. The maximum test duration was 900 s (15 min). The stopwatch was started immediately after the sweetcorn had been dropped in the cage and was stopped when a mouse was seen eating it for at least 2 s. Mice that did not consume any sweetcorn were scored the maximum latency (900 s).

### 2.7. Interaction with Handler’s Hand

**Figure 1:**
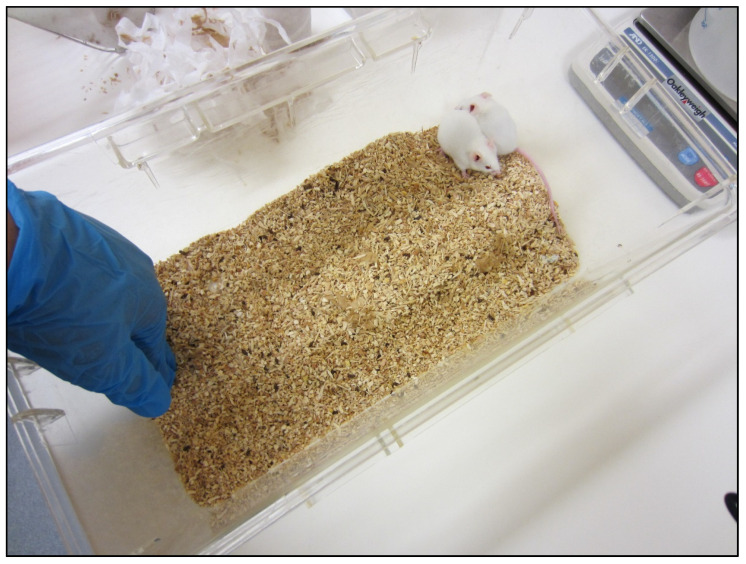
Handler interaction test set-up. The handler’s hand remained motionless for 3 min whilst the voluntary responses of the mice were recorded. The handler wore nitrile gloves, as during marking.

To assess mouse fear of the handler, we observed their voluntary interaction with the handler [8]. All mice were tested in the procedure room immediately after their physical health was inspected: 1 day after treatment, 1, 2, 3 and 4 weeks (9 a.m.–10 a.m.). The handler—who had marked the EP, EP+A and MP mice—opened the cage and inserted her hand into the front half of the homecage (Figure 1). The hand remained motionless against the near wall of the cage for 3 min. Every 15 s, a female observer (NC) recorded whether each mouse was in the half of the cage nearest to or furthest from the hand. The frequency of sniffing or chewing the glove, and defensive burying (pushing bedding material towards the hand) was also recorded.

### 2.8. Faecal Corticosterone Measurement

Faeces for corticosterone measurement were collected at three time points: 1 day before treatment procedure (baseline), 6 h after treatment, and again 4 weeks later, always between 3 p.m. and 4.15 p.m. Fresh faecal pellets were collected from the weighing beaker (following weighing 1 day before treatment) or from the EPM apparatus after testing, using a pair of clean forceps. 

Pellets were placed into a micro-centrifuge tube, and within 2 h were frozen at −80 °C. For each time point, pellets from three mice per treatment group were pooled together to obtain at least 0.05 g of faeces for corticosterone assay. Following this, 1 mL of 90% methanol was added to each sample. The samples were homogenised using a mechanical homogeniser for 20–30 s and mixed using a vortex at maximum speed for 30 min. The mixture was centrifuged for 10 min at 2000 rpm. The supernatant was extracted into a clean micro-centrifuge tube and dried in a Speedvac at 45 °C for 4 h. The dried samples were stored at −80 °C. 

On the morning of the assay, the dried samples were resuspended in 1 mL PBS (1× ) and mixed using a vortex mixer for 1 min. Corticosterone concentrations were determined using the IDS OCTEIA Corticosterone HS Enzyme immunoassay (Immunodiagnostic Systems Ltd., Boldon, UK) according to manufacturer recommendations. The absorbance of each well was measured at 450 nm (reference 650 nm) using a Tecan Sunrise microplate reader and Magellan software.

Corticosterone concentrations were quantified by interpolating a standard curve (Sigmoidal, 4PL curve) using GraphPad Prism 6 (GraphPad Software Inc., California, CA, USA). The value for each pooled sample (ng/mL) was then divided by its weight to obtain the corticosterone concentration per gram faeces (ng/g).

### 2.9. Statistical Analysis

Statistical analysis was carried out using IBM SPSS Statistics versions 22 and 26 (IBM, Portsmouth, UK). Linear mixed models were used for continuous data where residuals fitted the normal distribution, generalised estimating equations were used when data included many zeroes, and generalised linear mixed models for other binary data. For homecage behaviour models, Treatment and the order in which videos were observed were fixed effects, and Cage was the random factor. For the elevated plus maze and the hand-interaction test, Treatment and Week were fixed effects while Cage was random. The interaction between Treatment and Week was tested, and if not significant, it was removed and the main effects tested. For corticosterone, Treatment and Timepoint and their interaction were fixed, whilst the pooled faecal samples were random effects. 

The distribution pattern of all continuous data was checked by visual inspection of residual histograms and Q-Q plots, and by carrying out Kolmogorov–Smirnov and Shapiro–Wilk tests of normality. Continuous data not fitting the normal distribution were log, square-root or inverse square-root transformed and rechecked for normality. Whether or not mice ate the novel food item were analysed using Fisher’s exact test. Kruskall Wallis tests were used for non-normal count data that were not repeated across time. When tests showed statistically significant overall treatment effects, post-hoc pairwise comparisons were used to reveal which treatments differed. 

A *p*-value of <0.05 was accepted as significant for most measurements, except for tests that generated multiple comparisons, so a more stringent threshold was used to account for the false discovery rate [37]. Specifically, 138 comparisons were made for the homecage analyses, and when the false discovery rate was controlled for, the statistical significance level was *p* < 0.018. Additionally, the Hand Interaction test generated 73 comparisons, so when the false discovery rate was corrected for, *p* < 0.007 was considered significant. Results with *p*-values between the FDR-corrected threshold and 0.05 are reported as nonsignificant trends for completeness.

## 3. Results

### 3.1. Immediate Responses to Identification Marking

There were no significant treatment effects on the immediate behavioural responses of mice (Table 2).

**Figure 2:**
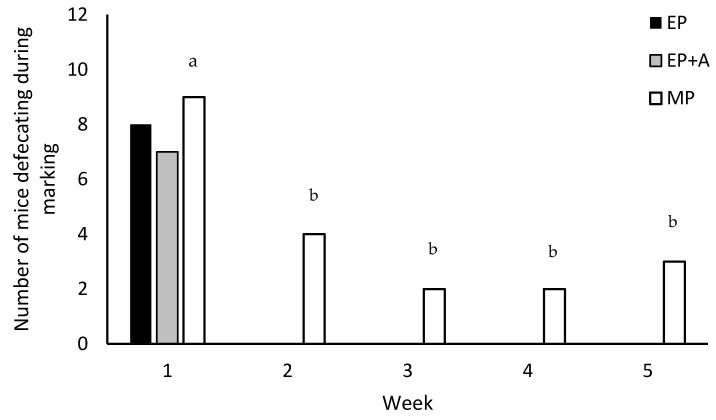
The number of mice defecating during different methods of identification marking. The treatments (*n* = 12) were as follows: EP = ear-punch; EP+A = ear-punch plus anaesthetic cream; MP = permanent marker on the tail. EP and EP+A was only conducted once per mouse, but MP was repeated weekly. MP mice were significantly more likely to defecate at ^a^ first application than at ^b^ subsequent applications (*p* < 0.001).

With repeated tail-marking, the MP mice defecated significantly more at the first tail-marking than during their four subsequent marking events (χ2 = 36.034; *n* = 12; *p* < 0.001; Figure 2). They did not vocalise during tail-marking, and there were no significant differences in the proportion of mice urinating over the five marking sessions (3–5 mice).

### 3.2. Body Weight and Physical Health

The total body weight gain over 4 weeks did not differ significantly (*p* = 0.945) between treatments. We observed no signs of barbering, scratches or wounds on any of the mice.

### 3.3. Homecage Behaviour after the Treatment Procedure

**Figure 3:**
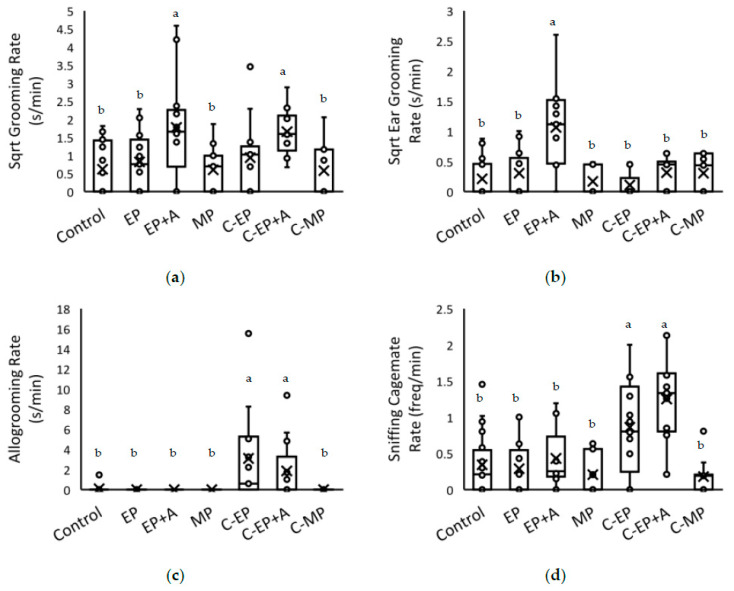
Immediate effects of identification marking methods on the homecage behaviour of mice and their cagemates. The behaviour was recorded for 5 min following identification marking of marked mice. General grooming of the head and body (box with bar = median +/− IQR; x = mean) is shown in (**a**); grooming of the ears in (**b**); allogrooming of the cagemate is shown in (**c**); sniffing of the cagemate is shown in (**d**). The treatments are as follows: Control = neither mouse was marked; EP = ear-punch; EP+A = ear-punch plus anaesthetic cream; MP = permanent marker on the tail; C-EP = cagemates of EP mice; C-EP+A = cagemates of EP+A mice; C-MP = cagemates of MP mice. ^a^ Significantly greater than ^b^ (*p* < 0.018, the significance threshold following controlling the false discovery rate).

Statistically significant results are presented in Table 3 (and see Supplementary Videos S1–S8 for examples of behavioural effects). EP+A mice groomed themselves, and their own ears, more than all other mice except C-EP+A and C-EP mice; C-EP+A mice also groomed themselves (but not specifically their ears) more than mice in the remaining treatments (Figure 3). The effect was quite large with, for example, EP+A mice spending a mean +/S.E. of 5.0 +/− 1.9 s/min grooming themselves versus the MP mice spending 1.1 +/− 0.3 s/min grooming themselves. There was no significant interactive effect of treatment on grooming of the right versus left ear, so ear-punched mice seemingly did not groom their punched ear more frequently than their unmarked ear (*p* = 0.074).

Both EP and EP+A mice were allogroomed and sniffed by their cagemates significantly more than any other groups (Table 3; Figure 3). The difference was stark, with 7/13 EP mice being allogroomed 19 times, 6/13 EP+A mice being allogroomed 12 times, whilst only 2/24 control mice allogroomed once each; no allogrooming occurred in the other treatments.

Facial grimaces were recorded significantly more in C-MP mice than in other mice apart from EP and EP+A (Table 3). After controlling for the false discovery rate, there was only a nonsignificant trend for grimacing to occur more in EP+A mice than in MP and C-EP mice. Whilst the highest proportion of mice grimacing was in C-MP mice (6/11 mice showing a total of 7 grimaces), the relatively few mice exhibiting them in the two ear-punch treatments tended to do so repeatedly (EP: 3/13 mice showing 10 grimaces; EP+A: 4/13 mice showing 11 grimaces). In other treatments, either zero or one grimace was observed.

There were no significant treatment effects on time spent in the tunnel, out of sight, rearing, eating/drinking, climbing, or freezing. ‘Other active’ behaviour showed a nonsignificant trend to be greater in Control mice than in EP mice, their cagemates, and C-EP+A (Table 3). The following behaviours were too rare for analysis. Aggression was only initiated by one Control and one C-EP mouse; grooming of a mouse’s own tail was seen in two EP mice and one Control; rapid shuttling was seen in one Control, one EP mouse, and one C-EP+A mouse; and body shaking was seen in three EP+A mice, two EP mice, and one C-EP+A.

The order in which videos were watched was associated with decreased recording of self-grooming (F_1,89_ = 8.216, *p* = 0.005) and sniffing of the cagemate (F_1,89_ = 9.703, *p* = 0.002) over time.

### 3.4. Elevated Plus Maze

There were no significant treatment effects in the EPM. We only found time effects, with significantly more head dips (F_1,160_ = 20.507, *p* < 0.001) and stretch attend postures (F_1,149_ = 11.615, *p* = 0.001) during the first test session than the second, but less urination (F_1,160_ = 8.280, *p* = 0.005) and defecation (F_1,158_ = 90.307, *p* < 0.001). Mice also spent less time in the central square (F_1,83_ = 28.202, *p* < 0.001) and more time in closed arms (F_1,77_ = 0.689, *p* < 0.001) during the first test session than the second.

### 3.5. Hyponeophagia Test

**Figure 4:**
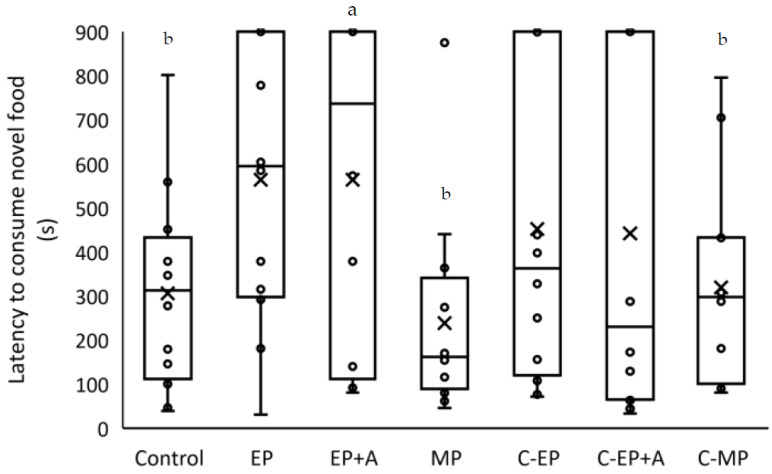
Effects of identification marking methods on latency to eat a novel food item. Data from all mice are displayed (box with bar = median +/− IQR; x = mean), including those who did not consume novel sweetcorn and thus attained the maximum latency of 900 s (*n* = 12/group). The treatments are as follows: Control = neither mouse was marked; EP = ear-punch; EP+A = ear-punch plus anaesthetic cream; MP = permanent marker on the tail; C-EP = cagemates of EP mice; C-EP+A = cagemates of EP+A mice; C-MP = cagemates of MP mice. ^a^ Significantly fewer mice consumed novel food than ^b^ (*p* = 0.001), but the latencies of mice who did consume food showed no significant treatment effects.

Significantly fewer EP+A mice (6/12 mice) ate the sweetcorn than the MP, C-MP and Control mice (12/12 mice each) (X^2^ = 19.288; DF = 6; *p* = 0.001). In the other treatments, 7/12 C-EP+A, 8/12 EP mice and 9/12 C-EP ate sweetcorn during the test. However, of the mice who did eat, the mean latency to start eating did not significantly differ between treatments (*p* = 0.230). Latency data from all mice, including those not eating the sweetcorn, are shown in Figure 4. 

### 3.6. Interaction with Handler 

**Figure 5:**
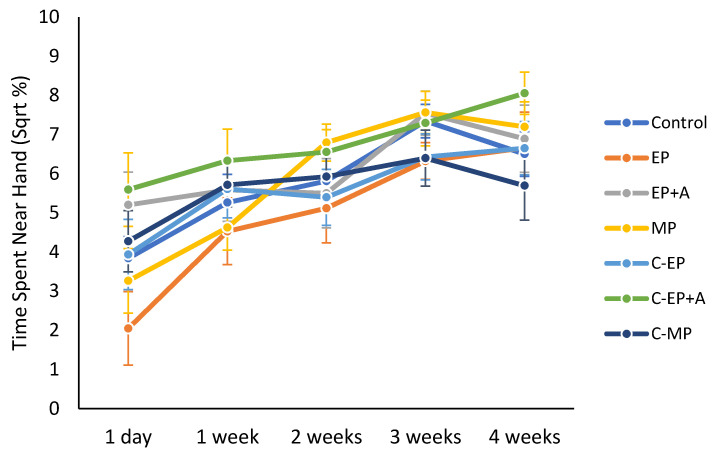
Effects of identification marking of mice on voluntary interaction with the handler’s hand. The hand interaction test was conducted one day following identification marking, and then weekly for the subsequent four weeks. The square-rooted mean +/− SE percentage of time the mice spent in the half of the cage nearest the hand is shown. The treatments are as follows: Control = neither mouse was marked; EP = ear-punch; EP+A = ear-punch plus anaesthetic cream; MP = permanent marker on the tail; C-EP = cagemates of EP mice; C-EP+A = cagemates of EP+A mice; C-MP = cagemates of MP mice. EP mice spent significantly less time than near the hand generally than C-EP+A mice (*p* = 0.006), and all mice spent more time near the hand as time progressed (*p* < 0.001).

Treatment significantly affected interaction with the handler (F_6,412_ = 3.055; *p* = 0.006), because EP mice spent significantly less time near the hand than the C-EP+A (Mean+/− S.E square root % time near hand for EP = 4.93 +/− 0.93; for C-EP+A = 6.76 +/− 0.72; *p* < 0.001; Figure 5). After correcting for the false discovery rate, there were also nonsignificant trends for the EP mice to spend less time near the hand than EP+A mice (*p* = 0.008) and MP mice (*p* = 0.036). The C-EP+A mice tended to spend more time near the hand than the other unmarked mice and controls (nonsignificant *p*-values ranging between 0.011 and 0.026).

There were nonsignificant trends for EP mice to sniff the hand less frequently than C-EP+A (F_6,412_ = 2.397; *p* = 0.027; post-hoc *p* < 0.001), and for MP mice to rear more frequently than EP+A mice (F_6,412_ = 2.666; *p* = 0.015; post-hoc *p* < 0.001). Touching the hand, sniffing the hand, rearing, and defensive burying showed no significant treatment effects.

As the weeks passed, mice across treatments spent more time near the hand (F_1,412_ = 69.974; *p* < 0.001; Figure 5), more frequently sniffed the hand (F_1,412_ = 111.281; *p* <0.001), touched the hand (F_1,412_ = 25.044; *p* < 0.001), reared (F_1,412_ = 34.873; *p* < 0.001) and showed more defensive burying (F_1,409_ = 16.825; *p* < 0.001). 

### 3.7. Faecal Corticosterone Analysis

Treatment showed no significant effects on corticosterone concentrations (*p* = 0.929). However, corticosterone concentrations were higher 6 h after treatment than at baseline (*p* < 0.001) and than 4 weeks after treatment (*p* = 0.036). At 4 weeks after treatment, faecal corticosterone level was also higher than the baseline (*p* = 0.030).

## 4. Discussion

We aimed to evaluate the efficacy of EMLA^TM^ cream in reducing any signs of pain and/or anxiety following ear-punching of weaned male BALB/c mice, if indeed any were seen, and the potential of tail-marking with permanent marker as a noninvasive alternative to ear-punching. Whilst most effects were nonsignificant, there were some behavioural changes following ear-punching without anaesthetic: half the EP mice pulled their heads away during marking; when back in the homecage, their cagemates allogroomed and sniffed them significantly more than in other treatments; EP mice consistently spent the least time near the handler in the handler interaction test. EMLA^TM^ application showed no obvious benefits over ear-punching alone, although EP+A mice did not avoid the handler to the extent that the EP mice did. The EMLA^TM^ may actually have worsened discomfort and anxiety as indicated by increased self-grooming of the head and body and of the ears in the homecage, and decreased consumption of novel food by EP+A mice the following day. There was an apparent contagion effect on grooming in the cagemates of EP+A mice. On the other hand, tail-marking appeared to have no adverse effects, with MP mice showing no significant differences from control mice in any test, although there was an unexpected effect on their cagemates, who showed more facial grimacing than other mice. These results will be discussed in more detail in turn.

### 4.1. Effects of Ear-Punching without EMLA^TM^

Half of the EP mice pulled their heads away during ear-punching, which is very similar to a related experiment [11], where approximately half of the 48 ear-punched mice pulled away versus significantly fewer (<5%) restraint-only mice. Pulling the marked body part away may signify an acute pain response [28].

Ear-punched mice, both with and without EMLA^TM^ cream, were allogroomed and sniffed significantly more by their cagemates than any other mice were in the 5 min following marking. The welfare implications of this are unclear. It is unlikely to have been due to the scent of the glove on the marked mice, because we did not see the same allogrooming in tail-marked mice. It also seems unlikely that the cagemates were attracted to any blood, because the attention was not always focused on the wound-site. Additionally, the allogrooming usually seemed gentle rather than vigorous [31], despite being persistent (see Supplementary Videos). Instead it seems more likely that the unmarked cagemates were responding to pain behaviour [38], ‘alarm’ odour(s) [26,39,40], and/or ultrasonic vocalisations [9] produced by the ear-punched mice. Mice can show prosocial or empathy-like ‘consolation’ behaviour in response to familiar conspecifics in pain, so this may be another example [41]. It is consistent with other observations that both mice and rats allogroom familiar conspecifics with experimentally induced pain [38,42].

We found no other effects of ear-punching on homecage behaviour, unlike Mazlan et al. (2018), where ear-punched mice showed increased freezing and ear-grooming relative to restrained and unhandled mice. Possible reasons for our lack of these homecage effects could be our smaller sample size, that our mice were paired (mice were singly housed in the previous study), and that we only observed behaviour in the 5 min following marking, whereas Mazlan repeated observations at intervals [43]. Ideally, further investigations would observe homecage behaviour for at least 15–20 min following handling [44].

The negative effects of ear-punching appeared to persist beyond the procedure itself, as indicated by the continued avoidance of the human hand by EP mice; the avoidance persisted for at least 2 weeks (Figure 5). This suggests that ear-punching may have produced a fear memory that could have increased stress during general handling in subsequent weeks. This result reached significance despite all the mice being lifted by the tail (the standard method in our laboratory at the time of the study); tail-lifting greatly reduces voluntary interaction with the hand compared with more physically supportive handling methods [8], so it is concerning that ear-punching was able to reduce voluntary interaction further still. We did not find significant effects of ear-punching on anxiety as measured via the EPM, hyponeophagia test, or faecal corticosterone, which could mean that the fear was specific to handler interactions, or perhaps could have been due to floor/ceiling effects produced by tail-lifting that masked any treatment effects [45]. Although we used several measures of anxiety, many more exist [46,47], so future studies could incorporate other tests and use more refined handling methods [8]. Additionally, here we could only use male BALB/c mice, so it would be worth assessing the effects of identification marking on different mouse strains and both sexes, ideally across different laboratories to maximise reproducibility [48].

We found no significant effect of ear-punching on aggression, unlike Gaskill et al. [12], because we saw almost no signs of aggression. This could be due to BALB/c siblings being used here, or because we used brief live scoring of aggression and wounding, rather than post-mortem pelt scores. Further research into the effects of identification marking should include greater monitoring of aggression, especially in aggressive mouse strains. We would recommend using more prolonged behavioural observation than used here [44], and post-mortem pelt-scoring as well as data from routine health checks [12,36].

### 4.2. Efficacy of EMLA^TM^ Cream

The application of EMLA^TM^ cream before ear-punching may have slightly reduced the proportion of mice pulling their head away during the procedure, and the avoidance of the hand in subsequent weeks, but neither reduction was statistically significant. EMLA^TM^ did not reduce the amount of allogrooming or sniffing from the cagemate once back in the homecage either, suggesting that any signals from the ear-punched mice were of a similar magnitude regardless of whether EMLA^TM^ was applied or not. There was a nonsignificant trend for EP+A mice to grimace more than MP mice and most unmarked groups, which could indicate pain [20,30] or disgust [49], if confirmed in future work. In summary, EMLA^TM^ cream appeared ineffective at reducing pain from ear-punching. 

We applied EMLA^TM^ cream 20 min before the procedure, whereas the manufacturer recommends 1 h (for human use) [15], and tMax in mice is about 30 min [50]. However, this is unlikely to explain the lack of efficacy, because 20 min was enough for EMLA^TM^ to effectively reduce pain signs in rabbits [16]. Additionally, EMLA^TM^ that was applied to mouse tails 1 h versus only 5 min before tail-biopsy was equally ineffective in preventing pain signs [19]. Thus, the current study is in accordance with the growing body of literature suggesting that EMLA^TM^ is not effective at reducing pain in mice and rats [17,18,19,20].

EMLA^TM^ cream caused some additional changes not seen with ear-punching alone. The increased ear grooming could have been due to tingling, numbing, itching or burning sensations (listed as possible side effects of EMLA^TM^ cream on the human patient leaflet [15]). Licking the paws after grooming the affected ear could also possibly numb the tongue and/or be distasteful. Ear grooming probably partially removed the cream before ear-punching, reducing its analgesic efficacy; we were unable to apply a dressing to the ear to prevent cream removal. The increased self-grooming may have been due to the altered sensation, or due to having the foreign substance on the body, or due to stress [51]. The unmarked C-EP+A mice also showed increased self-grooming. This could have been a behavioural contagion, stress-related or not, if they mirrored the grooming of their partner [38], or perhaps they acquired some cream when allogrooming their cagemate and were thus also attempting to remove it from their own bodies. 

The following day, the EP+A mice were significantly less likely to consume novel food than were MP, C-MP or control mice. It is worth noting that some of the EP, C-EP and C-EP+A mice also refrained from eating the food, whereas all the mice in other treatments consumed it. The reduced consumption could signify anxiety from the previous day [34], when ear-punching occurred and when EP+A mice were restrained twice: once for EMLA^TM^ application and once for ear-punching. Alternatively or additionally, perhaps the EP+A mice consumed some EMLA^TM^ cream, reducing their appetite the following day due to nausea. The potential toxic and other effects of mice ingesting small quantities of EMLA^TM^ cream are currently unclear. 

Given the apparent lack of efficacy of EMLA^TM^ cream in this context, it could be worth investigating in future whether any other analgesics or anaesthetics could minimise the negative effects of ear-punching without unwanted side-effects.

### 4.3. Tail-Marking Versus Ear-Punch

Tail-marked (MP) mice showed no significant differences from controls in any of the tests here, and they showed significantly fewer negative effects than EP and EP+A mice. They also showed no clear tail-directed behaviour back in the homecage. We did not find the significant reduction in EPM anxiety behaviour from tail-marking, or the increased aversion to handling, that was previously found in adult rats [21]. 

The mice habituated, rather than sensitised, to the tail-marking procedure over time, as indicated by the significant decrease in defecation during marking between the first and subsequent procedures. This is despite tail-lifting having been used, so the procedure could have been further refined if mice had been lifted via more physically supportive methods [8]. 

The unmarked C-MP mice were mostly unaffected by their cagemate being tail-marked, except that they grimaced significantly more in the 5 min following marking than all other mice apart from EP and EP+A mice. We did not hypothesise this effect, so it may be a Type I error (falsely significant). Alternatively, whilst we assumed that facial grimacing would signify pain [20,30] in EP and EP+A mice, perhaps the C-MP mice were instead grimacing because of the odour of fresh ink on their cagemate’s tail [11,21]; if so, it is unclear why the MP mice would not grimace similarly. The unexpected nature of this result calls into question the validity of our grimace observations as expressions of acute pain from ear incisions [52]; grimacing was not observed after ear-notching [20], but it was observed after ear-tagging [4]. Errors could have arisen because the footage was relatively wide-angle and filmed through the cage walls, so whilst narrowed eyes could clearly be seen, more subtle features were harder to visualise. This may have made it difficult to distinguish pain expression from, for example, expressions of distaste [49]. If the grimacing was due to aversion to the ink odour, this could have been transient, if the ink on the tail dried rapidly. 

Further research to discover which marker pen is least aversive and longest lasting, and what exact pattern of ink application to the tail is most tolerable to mice, would refine this marking method further still. Importantly, it will be crucial to ascertain whether any inks should be avoided due to potential toxicity [22] in this applied context, especially if used long term.

## 5. Conclusions

Whilst not all of our measurements showed significant treatment differences, there were some indications that ear-punching caused a degree of acute pain and moderately lasting aversion to the handler in 4–5 week old male BALB/c mice. We cannot recommend EMLA^TM^ cream for lessening the impact of ear-punching on mouse welfare, because it appeared to be ineffective in reducing pain and to cause some additional discomfort or anxiety. On the other hand, the results suggest that tail-marking using permanent marker is a more humane alternative to ear-punching if researchers or animal personnel are prepared to re-mark mice at appropriate intervals, and provided that no ear-tissue is needed from the mice at the point of identification-marking. Further investigations should include more in-depth behavioural observations and evaluations of ink and/or anaesthetic toxicity, using both sexes of mice and more than one strain. In the meantime, we recommend tail-marking mice for identification purposes, and encourage personnel to use the least aversive ink and handling methods possible when doing so.

## Figures and Tables

**Table 1:** Ethogram for coding homecage video recordings following treatment procedure. The ethogram was adapted from existing publications [20,30,31] and from pilot observations. The behaviours are listed in alphabetical order. Behavioural coding was done on one individual mouse at a time, so each video clip was observed twice to allow behavioural coding for both mice in a cage. Point events were recorded as frequencies only, whilst states were recorded as both frequencies and durations.

Behaviour	Behaviour Type	Description
Aggression	State	Mouse demonstrates one or more of the following behaviours: Chasing—focal mouse follows the same path as cagemate, cagemate is fleeing, both mice are running; Tail rattling—focal mouse waves tail side to side in fast almost vibrating motion; Biting—mouse attacks the cagemate with open mouth, appears to bite or latches on to recipient; Boxing/parrying/thrust—movements towards one another, kicking with forepaws.
Allogrooming	State	Focal mouse uses paws and/or mouth to brush, lick or nibble the fur of cagemate.
Body shake	Point event	Mouse rapidly shakes its whole body, as if to rid its coat of dust.
Climbing	State	Mouse hangs from the cage lid, with the forelimbs or all limbs gripping the lid bars.
Eating/Drinking	State	Mouse has its mouth touching food hopper or food pellet, with chewing movement of the jaw, or mouse has its mouth touching the water outlet.
Facial grimacing	Point event	Mouse demonstrates one or more of the following pain descriptors: Mouse shows orbital tightening/narrowing of the eyes (not in the context of grooming, because mice narrow/close their eyes to groom); Bulging on the bridge of the mouse’s nose and/or wrinkles on the side of the nose; Bulging of the cheeks; or Ears rotate outwards and/or backwards. Ears may fold to form a pointed shape. Space between the ear increases. (Whisker placement would normally also be included but it was not possible to see this level of detail in the footage.)
Freezing	State	Mouse makes no movement (except for slight head and/breathing movement), for at least 2 s.
Self-grooming (not including ears or tail)	State	Mouse demonstrates two or more of the following descriptors: 1. Mouse licks its forepaws; 2. Mouse makes repeated strokes along the snout with both forepaws; 3. Mouse makes semicircular movements over the top of its head and behind the ears with both forepaws; 4. Mouse licks or grooms its fur on its anterior part of the body and/or scratching with the hind paw; 5. Mouse licks or grooms its hind legs; 6. Mouse licks or grooms the genitals; 7. Mouse scratches any part of the body (other than the ears) with its hindpaw, a move isolated from the cephalocaudal grooming sequence.
Grooming left/right ear	Point event	Mouse strokes/scratches the left/right ear with its forepaw or hind paw.
Grooming tail	State	Mouse sniffs or licks its tail while holding it with both forepaws (isolated from the normal cephalocaudal grooming sequence).
In tunnel	State	Mouse has all four limbs in the shelter—behaviour is visible.
Not visible (tunnel)	State	Mouse is in the tunnel but detailed behaviour is not visible.
Not visible (other)	State	Mouse is located at a spot where it is hidden from scorer’s view (e.g., behind the food rack).
Other active behaviour	State	Collection of active behaviours not otherwise recorded, such as walking, sniffing, digging and gnawing.
Rapid shuttle	Point event	Mouse flits extremely fast from one location of the substrate to another.
Rearing	Point event	Mouse lifts its forepaws off the ground, mouse has its forepaws on the cage wall/shelter, only standing on its hind legs,
Sniffing cagemate	Point event	Mouse pauses and extends neck to touch cagemate with its nose. May also lightly touch the area with forepaws.

**Table 2:** Number of mice showing behavioural responses during treatment procedure. There were no significant differences between treatment groups (*n* = 12/group).

Response	Ear-Punch (*n* = 12)	Ear-Punch + EMLA^TM^ (*n* = 12)	Marker Pen (*n* = 12)	*p*-Value
Pulling head away	6	2	N/A	0.097
Vocalisation	0	1	0	N/A
Urination	4	3	4	>0.999
Defaecation	8	7	9	0.903

**Table 3:** Statistically significant effects and trends of identification marking methods on the homecage behaviour of mice and their cagemates.

Behaviour	Comparison	Effect Size	*p*-Value *
Allogrooming	Overall Treatment	H = 34.80	<0.001
	C-EP > Control	23.48 +/− 6.13	<0.001
	C-EP > EP	27.15 +/− 6.98	<0.001
	C-EP > EP+A	27.15 +/− 6.98	<0.001
	C-EP > MP	27.15 +/− 7.29	<0.001
	C-EP > C-MP	27.15 +/− 7.29	<0.001
	C-EP+A > Control	18.94 +/− 6.13	0.002
	C-EP+A > EP	22.61 +/− 6.98	0.001
	C-EP+A > EP+A	22.61 +/− 6.98	0.001
	C-EP+A > MP	22.61 +/− 7.29	0.002
	C-EP+A > C-MP	22.61 +/− 7.30	0.002
Ear grooming	Overall Treatment	F_6,89_ = 10.10	<0.001
	EP+A > Control	0.90 +/− 0.13	<0.001
	EP+A > EP	0.77 +/− 0.15	<0.001
	EP+A > MP	0.91 +/− 0.15	<0.001
	EP+A > C-EP	0.94 +/− 0.15	<0.001
	EP+A > C-EP+A	0.75 +/− 0.15	<0.001
	EP+A > C-MP	0.75 +/− 0.15	<0.001
Facial grimacing	Overall Treatment	H = 18.90	0.004
	EP+A > MP	15.23 +/− 7.50	0.042 ^FDR^
	EP+A > C-EP	15.23 +/− 7.18	0.034 ^FDR^
	C-MP > Control	21.41 +/− 6.66	0.001
	C-MP > MP	25.46 +/− 7.80	0.001
	C-MP > C-EP	25.46 +/− 7.50	0.001
	C-MP > C-EP+A	21.26 +/− 7.50	0.005
Other active behaviour	Overall Treatment	F_6,89_ = 2.22	0.048 ^FDR^
	Control > EP+A	0.13 +/− 0.05	0.010
	Control > C-EP	0.12 +/− 0.05	0.019 ^FDR^
	Control > C-EP+A	0.12 +/− 0.05	0.017
Self-grooming	Overall Treatment	F_6,89_ = 4.74	<0.001
	EP+A > Control	0.15 +/− 0.04	<0.001
	EP+A > EP	0.13 +/− 0.04	0.005
	EP+A > MP	0.16 +/− 0.05	0.001
	EP+A > C-EP	0.10 +/− 0.04	0.025 ^FDR^
	EP+A > C-MP	0.14 +/− 0.05	0.002
	C-EP+A > Control	0.13 +/− 0.04	0.001
	C-EP+A > EP	0.11 +/− 0.04	0.016
	C-EP+A > MP	0.14 +/− 0.05	0.003
	C-EP+A > C-MP	0.13 +/− 0.05	0.007
Sniffing cagemate	Overall Treatment	F_6,89_ = 13.39	<0.001
	C-EP > Control	1.02 +/− 0.21	<0.001
	C-EP > EP	1.09 +/− 0.24	<0.001
	C-EP > EP+A	1.04 +/− 0.24	<0.001
	C-EP > MP	1.32 +/− 0.26	<0.001
	C-EP > C-MP	1.02 +/− 0.25	<0.001
	C-EP+A > Control	1.31 +/− 0.21	<0.001
	C-EP+A > EP	1.38 +/− 0.24	<0.001
	C-EP+A > EP+A	1.32 +/− 0.24	<0.001
	C-EP+A > MP	1.60 +/− 0.25	<0.001
	C-EP+A > C-MP	1.31 +/− 0.25	<0.001

The behaviours were recorded for 5 min following identification marking of marked mice and are arranged in alphabetical order. The overall effect sizes are reported as H-values where Kruskall–Wallis tests were used for non-normally distributed data, and as F-values where linear mixed models were used; the post-hoc effect sizes were taken from model coefficients. FDR = these results are nonsignificant trends after adjusting the significance level to account for the false discovery rate, so they are reported for completeness. The treatments are as follows: Control = neither mouse was marked; EP = ear-punch; EP+A = ear-punch plus anaesthetic cream; MP = permanent marker on the tail; C-EP = cagemates of EP mice; C-EP+A = cagemates of EP+A mice; C-MP = cagemates of MP mice. * *p* < 0.018, the significance threshold following controlling the false discovery rate.

## Data Availability

The data presented in this study are available on request from the corresponding author. The data are not yet publicly available due to ongoing analysis by the authors.

## Supplementary Materials

The following are available online at https://youtu.be/8TTZF1j1fpg: Video S1. Homecage behaviour in Control Cage 16. Footage is shown from approximately 120 to 180 s. A variety of active behaviour and some rapid shuttling can be seen. https://youtu.be/JWq4G7mKWks: Video S2. Homecage behaviour in Control Cage 20. Footage is shown from approximately 60 to 120 s. The mice sniff the air and alternate between being in the tunnel and outside it. https://youtu.be/2i6bVcBT5-g: Video S3. Homecage behaviour in Ear-punch Cage 9. Footage is shown from approximately 140 to 200 s. The ear-punched (EP) mouse starts to the right-hand side of the unmarked cagemate (C-EP). The EP mouse shows a rapid shuttle and then freezing approximately 4 s into the clip before entering the tunnel, and the C-EP sniffs EP’s body from approximately 40 s into the clip. https://youtu.be/NTiYRxIY3Y8: Video S4. Homecage behaviour in Ear-punch Cage 37. Footage is shown from approximately 180 to 240 s. The ear-punched (EP) mouse starts in the foreground, closer to the camera than the unmarked cagemate (C-EP). The C-EP sniffs and licks the body and face of EP for much of the video. https://youtu.be/j3DzfStn2ks: Video S5. Homecage behaviour in Ear-punch + EMLATM Cage 14. Footage is shown from approximately 30 to 90 s. The ear-punched + EMLATM (EP+A) mouse starts in the tunnel, to the left of the unmarked cagemate (C-EP+A). The EP+A mouse grooms his face and the affected ear for much of the video, and C-EP+A occasionally sniffs EP+A. The facial expression with narrowed eyes was rarely recorded as grimacing because of its co-occurrence with grooming. https://youtu.be/TNDoYk3duZs: Video S6. Homecage behaviour in Ear-punch + EMLATM Cage 22. Footage is shown from approximately 100 to 160 s. The ear-punched + EMLATM (EP+A) mouse starts in the tunnel, to the left of the unmarked cagemate (C-EP+A). The EP+A mouse grooms and scratches at his face and the affected ear. The C-EP+A also grooms himself and then from approximately 30 s into the clip sniffs and licks the affected ear of EP+A. The facial expression with narrowed eyes was rarely recorded as grimacing because of its co-occurrence with grooming. https://youtu.be/dEpgBPkBOTQ: Video S7. Homecage behaviour in Marker pen Cage 7. Footage is shown from approximately 180 to 240 s. The mouse with marker pen on his tail (MP) starts to the left-hand side of the unmarked cagemate (C-MP). At approximately 8 s, C-MP sniffs MP, including his tail—this was the only instance of tail-sniffing observed in the study. https://youtu.be/kyA9FKkf-88: Video S8. Homecage behaviour in Marker pen Cage 27. Footage is shown from approximately 60 to 120 s. The mouse with marker pen on his tail (MP) starts in the tunnel, to the right-hand side of the unmarked cagemate (C-MP).
